# X-linked *RBBP7* mutation causes maturation arrest and testicular tumors

**DOI:** 10.1172/JCI171541

**Published:** 2023-10-16

**Authors:** Jingping Li, Huimei Zheng, Jiaru Hou, Jianhua Chen, Fengbin Zhang, Xiaohang Yang, Fan Jin, Yongmei Xi

**Affiliations:** 1 Department of Reproductive Endocrinology and; 2 Division of Human Reproduction and Developmental Genetics, Reproductive Medicine Center, Women’s Hospital, Zhejiang University School of Medicine, Hangzhou, China.; 3 Institute of Genetics, Zhejiang Provincial Key Laboratory of Genetic & Developmental Disorders, Zhejiang University School of Medicine, Hangzhou, China.; 4 Department of Pathology, Reproductive Medicine Center, Women’s Hospital, Zhejiang University School of Medicine, Hangzhou, China.

**Keywords:** Reproductive Biology, Genetic diseases

## Abstract

Maturation arrest (MA) is a subtype of non-obstructive azoospermia, and male infertility is a known risk factor for testicular tumors. However, the genetic basis for many affected individuals remains unknown. Here, we identified a deleterious hemizygous variant of X-linked retinoblastoma-binding protein 7 (*RBBP7*) as a potential key cause of MA, which was also found to be associated with the development of Leydig cell tumors. This mutation resulted in premature protein translation termination, affecting the sixth WD40 domain of the RBBP7 and the interaction of the mutated RBBP7 with histone H4. Decreased BRCA1 and increased γH2AX were observed in the proband. In mouse spermatogonial and pachytene spermatocyte-derived cells, deprivation of *rbbp7* led to cell cycle arrest and apoptosis. In *Drosophila*, knockdown of *RBBP7/Caf1-55* in germ cells resulted in complete absence of germ cells and reduced testis size, whereas knockdown of *RBBP7/Caf1-55* in cyst cells resulted in hyperproliferative testicular cells. Interestingly, male infertility caused by *Caf1-55* deficiency was rescued by ectopic expression of wild-type human *RBBP7* but not mutant variants, suggesting the importance of *RBBP7* in spermatogenesis. Our study provides insights into the mechanisms underlying the co-occurrence of MA and testicular tumors and may pave the way for innovative genetic diagnostics of these 2 diseases.

## Introduction

Non-obstructive azoospermia (NOA), characterized by failure of spermatogenesis, is present in 10%–15% of male infertility cases. This covers hypospermatogenesis, maturation arrest (MA), and Sertoli cell-only syndrome (1). MA is a common and diverse histopathological subtype of severe male infertility and presents with defects in germ cell proliferation and subsequent impairment of meiosis (2). Men with early MA (with mitotic/meiotic arrest) usually display small testes and increased levels of follicle-stimulating hormone (FSH), compared with late MA (with postmeiotic/spermiogenesis arrest) (3). The reported causes of MA are multifactorial and complex, including genetic factors, testicular torsion, varicocele, and congenital abnormalities (3). Genetic mutations of DNA damage repair genes such as *BRCA1*, which are known to cause MA (4), may also lead to testicular tumors (5).

Over the last few decades, there has been accumulating evidence that X-linked genes play a critical role in spermatogenesis. X-linked testis-expressed gene 11 (*TEX11*), for example, is sufficiently related to spermatogenic failure and can be used as evidence for the clinical diagnosis of NOA or oligozoospermia (6). Yatsenko et al. found that *TEX11* (MIM 300311) defects account for 15% patients with meiotic arrest (7). Hemizygous mutations of *TEX11* were also identified in 7 out of 479 patients with NOA (1.5%) in a Chinese Han population (6). Several other X-linked genes, including *GCNA* (MIM 301077), *MAGEB4* (MIM 300153), *HAUS7* (MIM 300540), *TAF7L* (MIM 300314), *RHOXF1/2* (MIM 300446, 300446), and *USP26* (MIM 300309), have also been reported as associated with azoospermia (8). Most recently, a large-scale analysis of the X chromosome in infertile men suggested that retinoblastoma-binding protein 7 (*RBBP7*) was the most frequently affected gene (9).

X-linked *RBBP7* is a ubiquitously expressed nuclear protein that contains a 6-repeat WD40 domain. Crystal structure and biochemical experiments showed human RBBP7 bound to histone H4 (10), where RBBP7 was found in the catalytic cores of histone deacetylase complexes that promote transcriptional repression of target genes, including those repressed by the retinoblastoma (11). Studies in human breast cancer cells have shown that RBBP7 interacts with the BRCT domain of the BRCA1 protein (12). In mouse oocytes, the depletion of *rbbp7* affects meiotic progression and chromosome segregation during meiotic maturation (13). In *Drosophila*, *Caf1-55* is the ortholog of *RBBP7* and forms a complex with histone H4 (14). Caf1-55 has been shown to function in the maintenance of germ stem cells (15).

In the present study, we performed whole-exome sequencing (WES) on DNA samples of 2 brothers with MA and their parents and mutation screening by means of Sanger sequencing of the *RBBP7* gene open reading frame. An identical hemizygous variant (NM_002893.4, c.1201ins1, p.W401M fs*4) was identified in the 2 brothers, occurring on chromosome Xp22.2 and affecting the last 2 exons of *RBBP7*. The function of *RBBP7* in spermatogenesis was further explored using in silico analysis, mouse germ cell lines, and *Drosophila* models. Our results suggest that X-linked *RBBP7* is crucial for spermatogenesis, and its deficiency can lead to inherited predisposition for MA, as well as the potential development of testicular tumors. Screening for X-linked *RBBP7* variants in azoospermic men could improve genetic counseling for, diagnosis of, and treatment of azoospermia.

## Results

### Clinical phenotypes.

**Figure 1:**
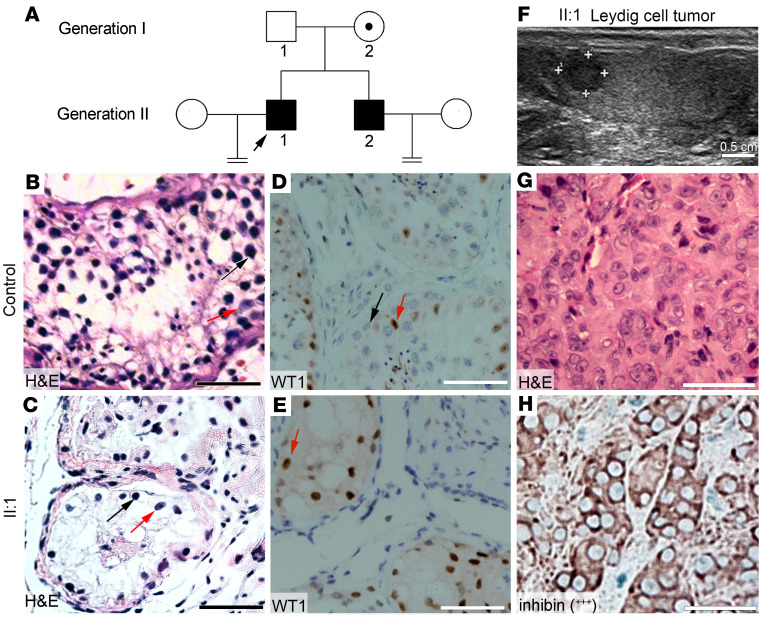
Clinical phenotypes. (**A**) Pedigree of the family with congenital azoospermia; an arrowhead indicates the proband, squares indicate men, circles indicate women, black symbols indicate affected individuals, unfilled symbols indicate unaffected individuals, and the obligate carrier is indicated using a dot-containing circle. H&E staining (**B**) and WT1 immunohistochemistry (**D**) analysis of cross sections of seminiferous tubules using testicular biopsy samples from a patient with obstructive azoospermia (as the control). H&E staining (**C**) and WT1 immunohistochemistry (**E**) analysis of cross sections of seminiferous tubules using testicular biopsy samples from II:1. (**B** and **C**) Red arrows indicate Sertoli cells and black arrows indicate spermatogonia. (**F**) Ultrasonography image shows a hypoechoic lesion (about 0.6 × 0.5 cm) in the upper pole of the right testis in the proband. (**G**) H&E staining analysis of cross-sections of Leydig cell tumor (LCT) from II:1. (**H**) Immunohistochemical staining of the tumor with inhibin of LCT from II:1. Scale bars: 50 μm.

An Asian family, including 2 brothers, was under study (Figure 1A). The proband (II:1) was a 27-year-old man in good health without obvious discomfort. His wife had not conceived naturally after 3 years of unprotected sexual intercourse. Within the past years, he had normal erectile function and ejaculation. The proband’s younger brother (II:2) was 26 years old, and his wife had not conceived naturally after 2 years (Figure 1A). The proband’s parents have had no reproductive health issues. The proband and his brother were conceived by natural means. The proband and his brother did not have a history of viral illness such as mumps, testicular trauma, long-term exposure to harmful substances such as heavy metals and radiation, or sexually transmitted infections such as gonorrhea, nongonococcal urethritis, and genital tuberculosis. They had had no instances of fevers, nor had they taken anabolic steroid or anti-androgenic drugs or undergone chemotherapy in the 3 months prior to surgery.

The proband and his brother had different testicle size. Ultrasonic examination showed that the patients’ mean testicle size was abnormally small, with 3.6 mL in the proband (II:1) and 4.5 mL in his brother (II:2). No obvious obstruction was found in their reproductive tract. The patients had normal karyotypes and no detected microdeletion of azoospermia factor regions on the Y chromosome. The proband underwent microdissection testicular sperm extraction (mTESE) at the Women’s Hospital, Zhejiang University School of Medicine. In detail, the mTESE surgery was performed under the surgical microscope (Carl Zeiss). Dissection of the testicular tissue was undertaken at 10×–20× original magnification in search for opaque and enlarged seminiferous tubules, which are more likely to contain spermatozoa. After the testicular tissue was thoroughly ground, a suitable quantity of fluid was extracted and observed under an inverted microscope (original magnification, ×400). No mature spermatozoa were retrieved after sufficient grinding of the testicular tissue. The proband’s brother (II:2) had also undergone mTESE in another hospital. No mature spermatozoa were able to be retrieved in his case also. The proband and his brother were not given any medications before sperm retrieval.

The cross sections of seminiferous tubules of biopsy samples were subjected to H&E staining (Figure 1, B and C) and immunostaining of Wilms’ tumor 1 (WT1) (Figure 1, D and E), a Sertoli cell marker. Results showed that Sertoli cells were obvious, but with only a few spermatogonia and no spermatocytes or sperm present in patient samples (Figure 1E), contrasting with the controls (Figure 1D). Serum sex hormone tests showed that the levels of FSH were higher than normal, with 45.5 IU/L in the proband (II:1) and 18.76 IU/L in his brother (II:2). The levels of luteinizing hormone (LH) were 27.3 IU/L in II:1 and 7.0 IU/L in II:2. The testosterone levels of the proband and brother were 6.5 nmol/L and 1.7 nmol/L, respectively, which indicated that the proband had a much higher level of testosterone than his brother. These tests showed that the symptoms of both brothers were consistent with those of early MA.

In addition, ultrasonography of the proband revealed a hypoechoic lesion (about 0.6 × 0.5 cm) in the upper pole of the right testis (Figure 1F). Ultrasound and H&E staining images showed that the boundary of the lesion was clear, and blood flow signal could be detected in the lesion (Supplemental Figure 1, A–C; supplemental material available online with this article; https://doi.org/10.1172/JCI171541DS1) (16). The levels of serum tumor markers, lactate dehydrogenase, alpha fetoprotein, and human chorionic gonadotrophin were in a normal range. CT scans showed no metastatic tumor in the pulmonary region or abdomen, with no evidence of abdominal, pelvic, or inguinal lymphadenopathy. The proband underwent microsurgical testicular tumor enucleation. H&E staining showed that the tumor tissue was composed predominantly of polygonal cells with abundant eosinophilic cytoplasm (Figure 1G). Immunohistochemical staining indicated intense and diffuse positive staining for inhibin (+++) (Figure 1H). The Ki67 proliferative index of the tumor was less than 5% (Supplemental Figure 1D). WT1 negativity suggested that the tumor was not derived from Sertoli cells (Supplemental Figure 1E). In addition, positive signals for vimentin (+) and calretinin (+) were observed (Supplemental Figure 1, F and G). These results indicate that this neoplasm is a benign Leydig cell tumor (LCT). After 1 year of follow-up, the proband’s health status was good, with no new testicular tumors observed.

### X-linked RBBP7 pathogenic variant suspected to cause MA and LCT.

We performed WES on DNA samples from these 2 brothers (II:1 and II:2) and their parents (I:1, I:2). For more than 93% of sequenced bases, the achieved quality score was higher than Q30. For each sample, the mapping efficiency of generated WES reads was more than 99.0%, and the coverage of exonic regions with at least 10 reads was more than 95.0%. The average sequencing depth on a target of 4 samples was 196.3 (197.0–202.4). In the exon region, I:1, I:2, II:1, and II:2 carried 23,210, 22,752, 23,980, and 23,416 variants, respectively. A total of 145 exon variants in 2 brothers were identical. These variants were filtered using a series of criteria (17). We finally focused on an X-linked *RBBP7* variant (NM_002893.4, c.1200dupA, p.W401Mfs*5), which has not been identified in population databases to our knowledge. This was predicted to be pathogenic by FATHMM-indel, CRAVAT, and SIFT_indels2 software.

**Figure 2:**
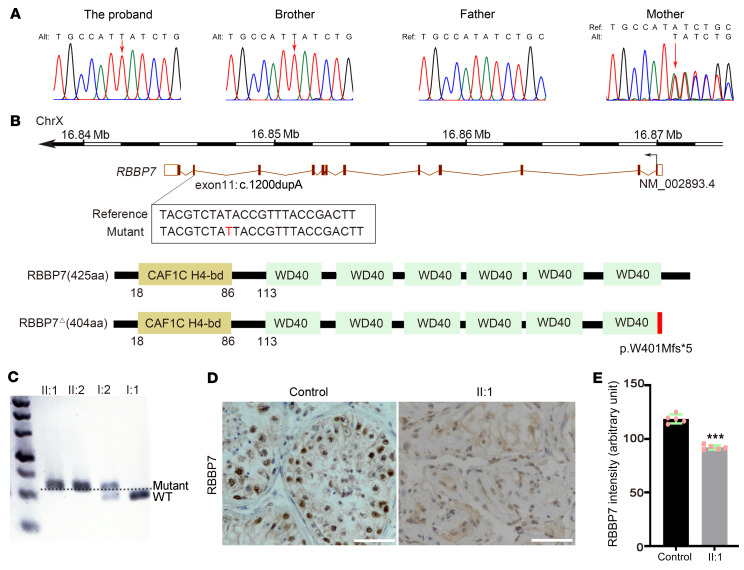
X-linked *RBBP7* pathogenic variant suspected to cause MA. (**A**) Sanger sequencing chromatograms validate the hemizygous *RBBP7* (NM_002893.4, c.1201ins1) missense variant in this family. The mother is a heterozygous carrier of the c.1201ins1 variation of *RBBP7*. The position of the variant is indicated by red arrows. (**B**) The upper panel shows the genomic structure of *RBBP7*, with 1 bp insert mutation mapped to isoform 1 (NM_002893.4, c.1201ins1). The lower panel shows the protein structure of the RBBP7 wild-type (425 aa) and RBBP7 mutant (RBBP7^Δ^) proteins. A missense mutation was present in the last WD40 domain of RBBP7. (**C**) RFLP analysis of affected brothers II:1 and II:2 and their mother and father. The PCR products were treated by the *Bgl* I restriction enzyme. Resulting fragments were separated by electrophoresis. The PCR product of the wild-type *RBBP7* was cut into a 19 bp fragment and a 207 bp fragment at 37°C, while the PCR product of the *RBBP7* mutation could not be cut. (**D**) RBBP7 immunohistochemistry analysis of cross sections of seminiferous tubules using testicular biopsy samples from a healthy control and II:1. (**E**) Quantification analyses of RBBP7 expression intensity; 5 random fields were selected for analysis. Data are shown as mean ± SEM. ***P < 0.001 by the unpaired, 2-tailed Student’s *t* test.

To evaluate the transmission pattern of the variant in the family, we performed Sanger sequencing analysis of *RBBP7* using DNA samples from the blood of family members. Patients II:1 and II:2 had hemizygous mutations of *RBBP7* with a single peak. Their mother (I:2) had a heterozygous mutation with a double peak (Figure 2A). The X-linked *RBBP7* mutation was a 1 bp inserted variant (Figure 2B). This leads to an earlier translation stop codon 4 amino acids from the insertion position at the last WD40 domain of the RBBP7 protein (p.W401Mfs*5) (Figure 2B). Restriction fragment length gene polymorphism (RFLP) analysis was then carried out to detect the mutation in the family and in 103 unrelated patients with NOA. The PCR product of the wild-type *RBBP7* could be cut into a 19 bp fragment and a 207 bp fragment, while the PCR product of the *RBBP7* mutation could not (Figure 2C). This *RBBP7* mutation was not observed in those 103 unrelated patients with NOA. In addition, a poly-antibody that recognizes all the amino acids of RBBP7 was used to identify the RBBP7-expressing cells of seminiferous tubules. We verified that RBBP7-positive spermatogonia, round spermatids, and Sertoli cells were distributed in the control but not in the patient (II:1) (Figure 2D), and the expression levels of RBBP7 were decreased in the patient, compared with the control (Figure 2E).

A quantitative analysis of single-cell RNA-sequencing data from human testicular cells, obtained via open databases, revealed high *RBBP7* expression in spermatogonia and spermatocyte cells, with lower expression levels in round and elongated spermatids (Supplemental Figure 2, A and B). Our immunostaining experiments verified these results (Supplemental Figure 2, C and D). Notably, RBBP7 protein was also detected in the Leydig and Sertoli cells (Supplemental Figure 2, C and D).

### The structural and functional impact of the p.W401M fs*5 mutation on RBBP7.

**Figure 3:**
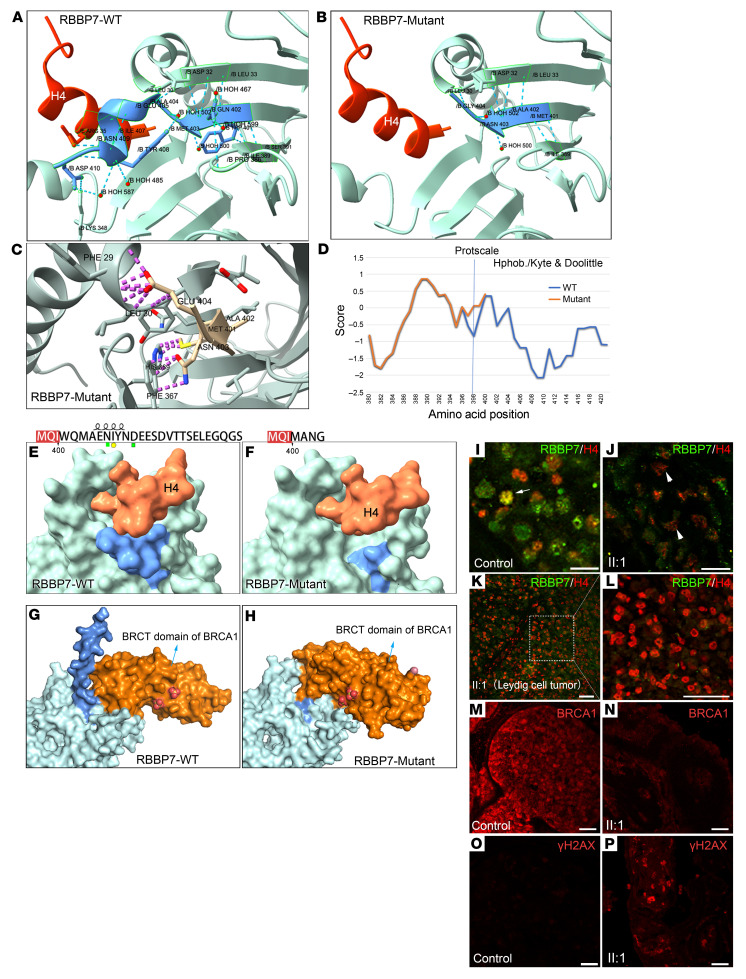
The structural and functional impact of the pW401M fs*5 mutation on RBBP7. (**A** and **B**) C-terminal of RBBP7. The graphics show the last WD40 domain of wild-type RBBP7 with 23 hydrogen bonds (shown with blue dotted lines) (**A**) and the mutant RBBP7 with only 8 hydrogen bonds (blue dotted lines) (**B**); the native structure (wild-type) (Protein Data Bank [PDB] code 7M3X and 3CFV). (**C**) View showing that the C-terminal structure of the mutant RBBP7 lacks the short C-terminal α-helix (Glu405 to Asn409) and leads to clashes with the nearby WD40 domain (purple dotted lines), in contrast with the wild-type RBBP7 (**A**). (**D**) Prediction of the hydrophobic character of the C-terminal of wild-type RBBP7 (blue) and mutant RBBP7 (orange) using the hydrophobicity/Kyte-Doolittle scale. (**E** and **F**) The structure of the wild-type RBBP7/histone H4 complex (**E**) and mutant RBBP7/histone H4 complex (**F**). The C-terminal of RBBP7 is shown in blue, and residues 25–41 in histone H4 are shown in orange. Yellow pentagon represents the key hydrophobic residues; green rectangles represent the key hydrophilic/charged residue. The spring-like structure represents the α-helix. The structure of the wild-type RBBP7/BRCA1 complex (**G**) and mutant RBBP7/BRCA1 complex (**H**). The C-terminal of RBBP7 is shown in blue, and the BRCT domain of BRCA1 is shown in orange. RBBP7/histone H4 immunofluorescence analysis of cross sections of seminiferous tubules using testicular biopsy samples from a healthy control (**I**) and from II:1 (**J**); RBBP7 (green) and histone H4 (red). (**K** and **L**) RBBP7/histone H4 immunofluorescence analysis of cross sections of LCT from II:1; **L** is an enlargement of panel **K**. BRCA1 immunofluorescence staining of cross sections of seminiferous tubules using testicular biopsy samples from a control (**M**) and from II:1 (**N**). γH2AX immunofluorescence analysis of cross sections of seminiferous tubules using testicular biopsy samples from a control (**O**) and from II:1 (**P**). Scale bar: 50 μm.

RBBP7 is a 6-repeat WD40 domain protein, consisting of a protruding N-terminal α-helix, a characteristic 7-bladed β-propeller structure, and a short C-terminal α-helix (18). We analyzed the impact of p.W401M fs*5 on RBBP7 in silico. A 3-dimensional (3D) modeling of the C-terminal region of wild-type RBBP7 revealed 23 hydrogen bonds (shown with blue dotted lines, Figure 3A). In contrast, the RBBP7 mutation affected the last β-propeller of the WD40 domain, resulting in only 8 hydrogen bonds and no short C-terminal α-helix (Figure 3B). This mutation led to steric clashes with the nearby amino acids (Figure 3C, purple dotted lines).

To detect hydrophilic and hydrophobic profiles of mutant RBBP7, we employed the hydrophobicity/Kyte-Doolittle scale and found that mutant RBBP7, as a truncated protein, had lost its C-terminal hydrophilic region (negative value) (Figure 3D, orange line). RBBP7 is known to interact with histone H4 when mediated by hydrogen bonds, salt bridges, and van der Waals contacts (10). The loss of the RBBP7 short hydrophilic/charged C-terminal α-helix, which sits on top of and extends the RBBP7 N-terminal α-helix, affects its interaction with the key residue Arg35 of histone H4 (Figure 3, E and F). RBBP7 has also been reported to interact with BRCA1 (12). We modeled the docking of BRCA1 and RBBP7. The result showed a decrease in the energy (–9.5 kcal/mol) of the BRCT domain of BRCA1 interacting with mutant RBBP7, compared with that with wild-type RBBP7, which shows the energy of –10.9 kcal/mol (Figure 3, G and H). Based on these data, we propose that the p.W401M fs*4 in RBBP7 could alter its bonding ability with its interacting proteins, including histone H4 and BRCA1, consequently affecting its biological function.

We then performed immunofluorescence staining with a monoclonal antibody raised against the full length of RBBP7 and a polyclonal antibody raised against the N-terminal of histone H4 to detect their colocalization in the seminiferous tubules. The colocalization of RBBP7 with condensed histone H4 signals was clearly observed in some of the testicular cells of the control (Figure 3I). However, in the sample of the proband, the signals of RBBP7 remained weak and associated with loosely distributed histone H4 signals (Figure 3J). In addition, RBBP7 signals were hardly detectable in the LCT of the proband, while histone H4 remained abundantly distributed (Figure 3, K and L). We also tested the BRCA1 expression in seminiferous tubules and testicular tumor tissue and found that BRCA1 was obviously decreased in the proband (Figure 3, M and N). BRCA1 is known to function in DNA damage sites, promoting DNA double-strand break (DSB) repair (19), which is essential for maintaining genomic stability. We checked γH2AX, a marker of DSBs. A higher level of γH2AX in the seminiferous tubule of the proband was detected, compared with the control (Figure 3, O and P).

### Rbbp7 deprivation affects the proliferation, apoptosis, and cell cycle of mouse germline cells.

Alignment of RBBP7 with its homologs in other organisms, including *Homo sapiens*, *Mus musculus*, *Danio rerio*, and *Drosophila melanogaster*, revealed the extensive conservation of protein sequences. Among these, human RBBP7 shares 99% amino acid similarity with mouse rbbp7 and 96% with *Drosophila* Caf1-55 (Supplemental Figure 3A). The 3D structure analysis showed the Caf1-55/histone H3 complex structure to be similar to the RBBP7/histone H4 complex structure (Supplemental Figure 3, B and C).

**Figure 4:**
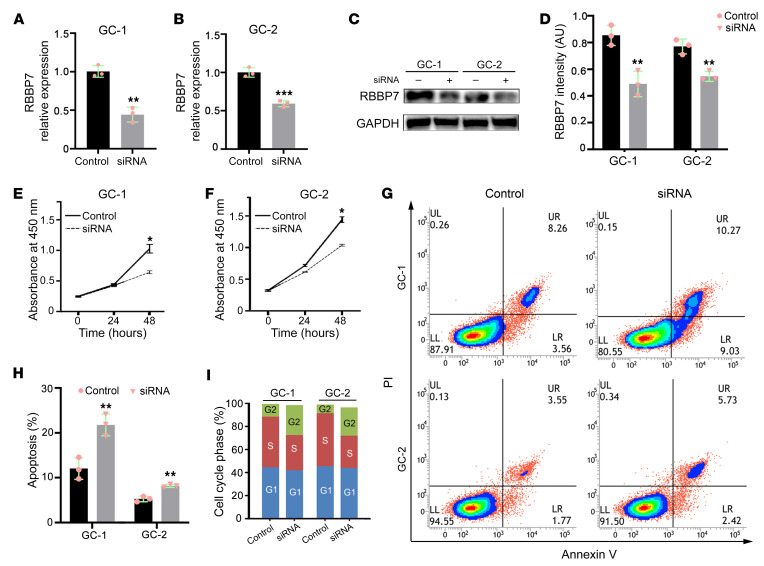
Deprivation of *rbbp7* affects the proliferation, apoptosis, and cell cycle of mouse GC-1 and GC-2 cells. (**A** and **B**) Relative mRNA levels of *rbbp7* in the negative control (NC) and si-*rbbp7*–treated GC-1 (**A**) and GC-2 (**B**) cells to validate knockdown efficiency. (**C**) Western blot detected by anti-rbbp7 and anti-GAPDH antisera on lysates of NC and siRNA-treated GC-1 and GC-2 cells. (**D**) Quantification analyses of rbbp7 in **C**. (**E** and **F**) Cell Counting Kit-8 (Biosharp) test in NC and siRNA-treated GC-1 (**E**) and GC-2 cells (**F**). (**G**) Cell component analysis in NC and siRNA-treated GC-1 and GC-2 cells by flow cytometry. (**H**) Percentages of apoptotic cells in NC (*n* = 3) and siRNA-treated (*n* = 3) GC-1 and GC-2 cells. (**I**) Flow cytometry analysis for cell cycle distribution of NC and siRNA-treated GC-1 and GC-2 cells. Two-tailed unpaired Student’s *t* tests were used for the statistical analysis. *P < 0.05; **P < 0.01; ***P < 0.001. All experiments were repeated 3 times.

We examined the cellular function of RBBP7 using 2 mouse germ cell lines, the spermatogonia cell line GC-1 and the pachytene spermatocyte-derived cell line GC-2. A small interfering RNA (*rbbp7* siRNA) was used to silence the endogenous mRNA expression of *rbbp7*. Quantitative reverse transcription PCR (qRT-PCR) and Western blot analyses showed that both mRNA and protein levels of rbbp7 were downregulated in the *rbbp7*-deprived GC-1 and GC-2 cells (Figure 4, A–D). We analyzed the cellular characteristics upon *rbbp7* deprivation and found that the cell proliferation of *rbbp7*-deprived cells was reduced in both GC-1 and GC-2 cells (Figure 4, E and F). Flow cytometry analysis revealed that a higher apoptosis rate of the *rbbp7*-deprived cells was also noted, as compared with the siRNA-negative groups (Figure 4, G and H). The average percentage of cells in S phase was decreased in the si-*rbbp7* groups (31.2% in GC-1 cells and 28.4% in GC-2 cells) compared with the siRNA-negative groups (43.9% in GC-1 cells and 46.3% in GC-2 cells) (Figure 4I). The ratio of cells in G2/M phase increased in the si-*rbbp7* groups (27.2% in GC-1 cells and 25.3% in GC-2 cells) compared with the siRNA-negative groups (10.3% in GC-1 cells and 7.8% in GC-2 cells) (Figure 4I and Supplemental Figure 4), indicating that silencing *rbbp7* led to cell cycle arrest at the G2/M phase. These results suggest that *rbbp7* is involved in the regulation of the proliferation, apoptosis, and cell cycle in mouse GC-1 and GC-2 cells.

### RBBP7/Caf1-55 deficiency leads to male infertility and small testis in Drosophila.

**Figure 5:**
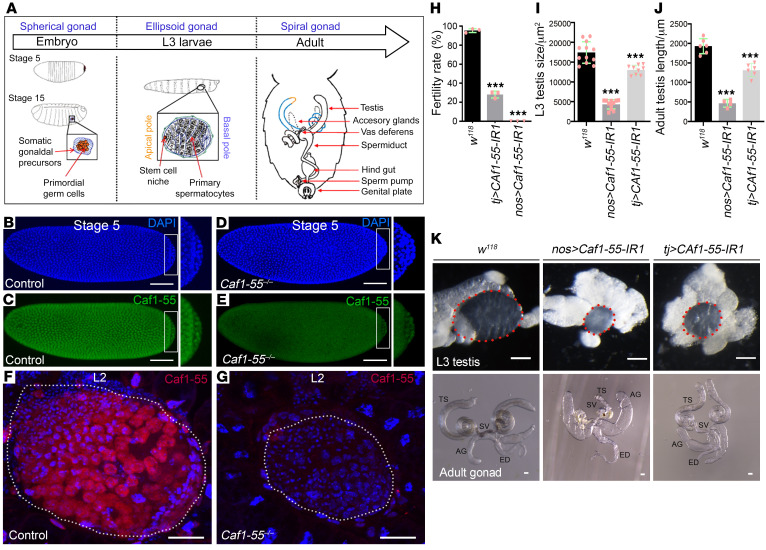
*RBBP7/Caf1-55* deficiency leads to male infertility in *Drosophila*. (**A**) *Drosophila* testis development begins from the embryonic stage. Pole cells are formed at the posterior of the embryo at stages 4–5, then migrate and differentiate to form the spherical embryonic gonads at embryonic stage 14. The gonads become ellipsoid at the larval stage and finally become incorporated into spiral tubules at the adult stage. (**B** and **C**) The expression pattern of Caf1-55. Caf1-55 was universally expressed in the nucleus of embryonic cells, including pole cells. (**D** and **E**) The maternal expression of Caf1-55 can be observed at the embryonic stage 5 of *Caf1-55^–/–^* animals. (**F** and **G**) Caf1-55 was detected at the second instar larval stage (L2) of the wild-type (**F**) but not in *Caf1-55^–/–^* animals (**G**). (**H**) Graph showing male fertility rate in different genotypes. (**I** and **J**) Larval (**I**) and adult testes’ (**J**) size from wild-type, *nos-Gal4*–driven *Caf1-55* IR1, and *tj-Gal4*–driven *Caf1-55* IR1 animals. (**K**) Images showing the testes of control and *Caf1-55* IR1 animals driven by *tj-Gal4* and *nos-Gal4* at the late third instar larval stage (upper) and from 5-day-old adults (lower). Scale bars: 60 μm. Data are shown as mean ± SEM. ***P < 0.001 by unpaired, 2-tailed Student’s *t* test. TS, testis; SV, seminal vesicle; AG, accessory gland; ED, ejaculatory duct.

To investigate the in vivo function of *RBBP7* in spermatogenesis, we employed a *Drosophila* model (Figure 5A). *Drosophila* testis development starts from the embryonic stage, and pole cells are formed at the posterior of the embryo at stages 4 to 5 (20). These migrate and differentiate to form the spherical embryonic gonads at embryonic stage 14. The gonads then become ellipsoid at the larval stage and finally incorporate into spiral tubules in the adult (Figure 5A). We performed immunostaining to systematically analyze the expression pattern of Caf1-55. Caf1-55 was universally expressed in the nuclei of embryonic cells, including pole cells (Figure 5, B and C). In *Caf1-55*–null (*Caf1-55^–/–^*) mutants (15), the maternal expression of Caf1-55 was detectable at embryonic stage 5 (Figure 5, D and E) but barely detected in the testis at the second larval stage (L2) (Figure 5, F and G). The mutants also showed a smaller sized testis, as compared with that of wild-type (Figure 5, F and G). It is notable that a high expression of phosphorylated histone H2Av (γH2Av) was detected in the testis of *Caf1-55* mutants at L2, but not in the wild-type (Supplemental Figure 5A), suggesting that DSBs had occurred because of loss of *Caf1-55*.

Because the *Caf1-55* mutants died at L2, we applied 2 *Caf1-55* RNAi lines (*Caf1-55 IR1*, *Caf1-55 IR2*) to assess the effects of *Caf1-55* deficiency on testis development. Two drivers, *tj-GAL4* expressed in somatic cyst stem cells and cyst cells (analogous to mammalian Sertoli cells) and *nos-GAL4* expressed in early germline cells (including germ stem cells), were used to achieve the cell-specific knockdown of *Caf1-55* in *Drosophila* testis (Supplemental Figure 5, B and C, and Supplemental Figure 6, A and B). A fertility test showed that wild-type females, when crossed with males from 2 RNAi lines driven by either *tj-GAL4* or *nos-GAL4* males, laid similar numbers of eggs as those crossed with the wild-type males. However, the percentage of hatched embryos derived from *Caf1-55*–knockdown males was remarkably decreased (Figure 5H and Supplemental Figure 6C), indicating male sterility.

We examined the gonads of *Caf1-55* deficiency males at third instar larval stage (L3) and adult stage. The morphology of the gonads was ellipsoid, but the size of gonads was much smaller in the *Caf1-55*–knockdown animals than in those of the control males at L3 (Figure 5, I and K, and Supplemental Figure 6). Statistical analysis showed a 73.53% reduction in the testis size for *nos>Caf1-55* RNAi males and a 21.76% reduction for *tj>Caf1-55* RNAi males at L3 (Figure 5I and Supplemental Figure 6M). In the adult stage, *nos>Caf1-55* RNAi males showed extremely tiny testes (with a 73.68% reduction) and completely empty seminal vesicles (Figure 5, J and K, and Supplemental Figure 6). *tj>Caf1-55* RNAi resulted in testes of slightly reduced size (with a 28.95% reduction) and almost empty seminal vesicles (Figure 5, J and K, and Supplemental Figure 6). These observations suggest that lack of *Caf1-55* in either germ cells or somatic cells leads to small testes.

### Germ cell absence and testicular cell hyperproliferation upon Caf1-55 knockdown.

**Figure 6:**
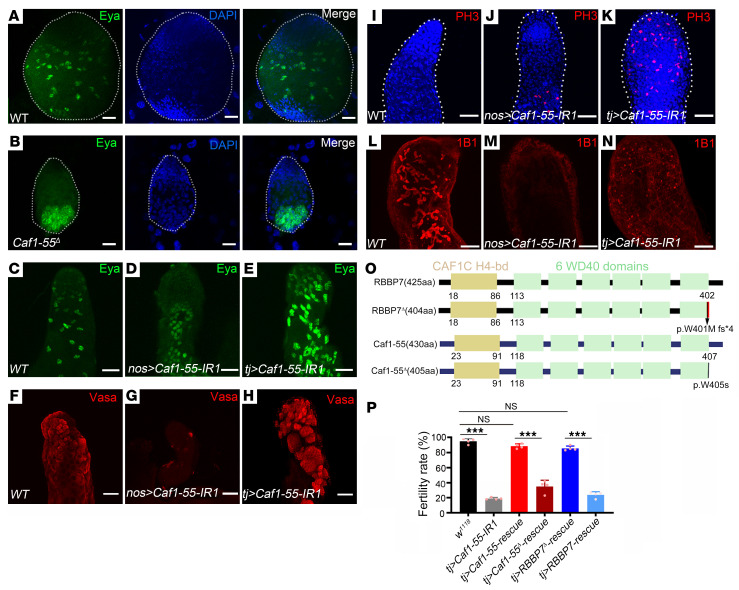
Loss of *Caf1-55* leads to testicular tumors in *Drosophila*. (**A** and **B**) Eya immunofluorescence analysis of larval testes of wild-type (**A**) and *Caf1-55^–/–^* animals (**B**). Immunostaining of testis with Eya (**B**–**E**), Vasa (**F**–**H**), PH3 (**I**–**K**), and 1B1 (**L**–**N**) from different animal genotypes. (**O**) Schematic representation of the human RBBP7 wild-type (425 aa) and RBBP7 mutant (RBBP7^Δ^, 404 aa) proteins and the *Drosophila* Caf1-55 (430 aa) and Caf1-55 mutants (Caf1-55^Δ^, 405 aa). (**P**) Graph showing male fertility test among different genotypes where *y* axis represents the embryo hatching rate. The male fertility is 0 in *nos-Gal4*–driven *Caf1-55*–knockdown animals and is about 25%–30% in *tj-Gal4*–driven *Caf1-55*–knockdown animals. The male fertility rate could be largely rescued by overexpressing wild-type *RBBP7* or *Caf1-55*, but not with mutant *RBBP7* or *Caf1-55*, in *tj-Gal4*–driven *Caf1-55*–knockdown animals. All experiments were repeated 3 times (each, *n* = 60). Scale bars: 50 μm. Data are shown as mean ± SEM. ***P < 0.001 by 1-way ANOVA followed by Bonferroni’s post hoc test.

To determine the effects of *Caf1-55* knockdown on *Drosophila* testis, we selected several protein markers to characterize the specific cell types. Immunofluorescence staining with eyes absent (Eya), a mature cyst cell marker, and Vasa, a germ cell marker, showed that at the L2 testis of *Caf1-55^–/–^* mutant males, cyst cells accumulated as tumor-like (Figure 6, A and B). The adults of both *nos>Caf1-55* RNAi flies and *tj>Caf1-55* RNAi flies also showed an increase in cyst cells, compared with the controls (Figure 6, C–E). In addition, *nos>Caf1-55* RNAi males showed complete loss of germ cells (Figure 6G), while *tj>Caf1-55* RNAi resulted in germ cells’ overproliferation (Figure 6H). We further performed immunostaining of PH3, a marker for proliferating cells. In wild-type testis, a small number of mitotic germ cells were seen near the tip of the testis (Figure 6I and Supplemental Figure 7A, white arrow), and more distally along the testis, a single PH3-positive meiotic cyst could be observed (Supplemental Figure 7A, red arrow). However, in *nos>Caf1-55* RNAi and *tj>Caf1-55* RNAi testis, several groups of mitotic cells were seen at the tip, middle, and distal end of the testis (Figure 6, J and K; Supplemental Figure 7A, green and yellow arrows; and Supplemental Figure 7B).

During spermatogenesis, spermatogonia and spermatocytes contain spectrin-rich structures known as fusomes (21), which can be visualized by immunostaining with 1B1, a fusome marker. In the wild-type testis, 1B1 signal displayed pointed and branched fusomes (Figure 6L), but no 1B1 signal was detected in *nos>Caf1-55* RNAi testis (Figure 6M and Supplemental Figure 7). In *tj>Caf1-55* RNAi testis, increased pointed fusomes and decreased branched fusomes were observed (Figure 6N, Supplemental Figure 7), indicating an accumulation of undifferentiated germ cells.

### RBBP7 rescues male infertility caused by Caf1-55 deficiency in Drosophila.

To investigate whether *RBBP7* could rescue fertility in *Drosophila* males with loss of *Caf1-55*, we generated transgenic flies that carried either a wild-type human *RBBP7* or a mutant human *RBBP7*, as well as a mutant *Caf1-55* line and an overexpressing wild-type *Caf1-55* line (15), to test genetic rescue (Figure 6O). Results showed that in the presence of ectopic wild-type human *RBBP7* or *Caf1-55*, the rate of male fertility was increased in *tj>Caf1-55* RNAi animals (Figure 6P), whereas in the overexpression of mutant human *RBBP7* or mutant *Caf1-55* in *tj>Caf1-55* RNAi, no such rescue was observed. These data suggest that normal human *RBBP7* rescues male infertility in *Caf1-55*–deficient *Drosophila*, while *RBBP7* mutation fails to rescue.

## Discussion

Spermatogenesis is a highly complex process that includes unique transcriptional regulation and massive chromatin alterations required for mitosis, meiosis, and postmeiotic maturation (22). As an important component of many chromatin modifier complexes (23), RBBP7 participates in transcriptional repression and chromosome remodeling, thus regulating cell proliferation, differentiation, and apoptosis (11). In several cancers, knockdown of *RBBP7* inhibits cell proliferation and metastasis (24). Expression profiling of rat male meiosis and gametogenesis has uncovered *Rbbp7* as a candidate factor involved in meiotic division (25). The involvement of RBBP7 in testicular tumors has not been previously reported to our knowledge.

Leydig cell hyperplasia (LCH) is common among men with NOA. LCH can occur with an increased number of Leydig cells that display intertubular growth patterns and form multiple clusters (26). This differs from the LCT that usually presents as a well-defined, solitary nodule (26, 27). Our ultrasound examination results and testicular pathological examination both indicate the characteristics of LCT in the proband, showing a clear boundary of the tumor lesion with no seminiferous tubules inside but with the blood flow signal detected (Supplemental Figure 1, A–C). These data helped us differentiate that this lesion represented an LCT rather than LCH.

The hormonal phenotypes are different between brothers. The testosterone (T) levels in the proband were higher before surgery than after the removal of LCT. Decreases in T levels were observed at the 3-month and 20-month periods postsurgery (Supplemental Table 3). These results suggested that LCT may have had an effect on the production of T. The proband’s brother had low level of T, but also with low level of LH, compared with the proband. There may be several explanations. First, the proband and his brother, one with LCT, another without, are not twins; thus, some level of genetic heterogeneity is likely. Second, in the negative feedback regulation, T associated with dihydrotestosterone directly suppresses the pituitary secretion of LH, whereas estradiol (E2) suppresses LH by inhibiting gonadotropin-releasing hormone secretion from the hypothalamus (28). In our study, E2 level was much lower in the proband but normal in his brother (Supplemental Table 3). Third, LH secretion is affected by prolactin (PRL). Hyperprolactinemia causes decreased T secretion and low serum T concentrations, also with low LH and FSH secretion in infertile men (29, 30). We found that serum PRL concentration of the proband’s brother was higher than that of the proband (Supplemental Table 3). This may contribute to the lower levels of T, LH, and FSH in the brother.

Conditional knockout of *Brca1* in mouse germ cells results in smaller testes, a large reduction in germ cells, an absence of meiotic cells, and abnormal differentiation of spermatogonia (31). In our study, the lack of *rbbp7* in GC-1 and GC-2 cell lines caused the inhibition of both the cell cycle and proliferation and the promotion of apoptosis. The investigated variant here in human *RBBP7* resulted in the loss of the C-terminal hydrophilic region, affecting its interaction with proteins such as histone H4 and BRCA1. Interestingly, BRCA1 binding to RBBP7 occurred at the same docking pocket where histone H4 binds to RBBP7. In LCT specimens of the proband, low levels of BRCA1 and RBBP7 were observed, leading to an impaired DSB repair system and increased risk for testicular tumor development. Knockdown of *Caf1-55* in *Drosophila* somatic cyst cells also resulted in high DSB levels and overproliferated testicular cells. Thus, the concurrent presence of male infertility and LCT may be linked to *RBBP7* mutations affecting key genome stability factors, such as histone H4 and BRCA1. Further research is needed to fully understand the mechanisms underlying this relationship and to develop effective treatments.

Riera-Escamilla et al. identified *RBBP7* mutations in 10 out of 2,354 men (0.42%) with azoospermia/cryptozoospermia (9), but their studies did not find the *RBBP7* mutation that we describe in the present study. Our systematic investigations from *Drosophila* embryo to adult revealed that RBBP7 has different roles in germ cells and somatic cells. Knockdown of *Caf1-55* in early germline cells leads to absence of spermatogenic cells, while the lack of *Caf1-55* in testicular somatic cells results in abnormal proliferation and differentiation in both germline cells and somatic cyst cells. Inactivation of Caf1-55/RBBP7 in either germline cells or cyst cells in *Drosophila* resulted in small testes and spermatogenic failure, resembling the phenotypes of *RBBP7* mutations in human patients. Ectopic expression of human wild-type *RBBP7*, rather than the mutant *RBBP7*, could rescue *Caf1-55*–linked male infertility.

In conclusion, we identified a deleterious hemizygous variant of the X-linked RBBP7 gene. The mutant type of RBBP7 protein may disrupt its interactions with histone H4 and BRCA1. Our results suggest that X-linked *RBBP7* is crucial for spermatogenesis, and its deficiency can lead to inherited predisposition to MA, as well as the potential development of testicular tumors. We and others (9) both have found that the mutations of *RBBP7* gene led to spermatogenesis defects and therefore could be a pathological cause for male infertility. However, the mutation related to both MA and LCT in our study was not found to be of prevalence. Further studies are required to verify the frequency of pathogenic variants of *RBBP7* in patients with NOA, and special attention should be paid toward screening for possible *RBBP7* mutation–related testicular tumors. It could be cost-effective to establish a panel of *RBBP7* combined with other azoospermia-associated gene variants for genetic diagnoses of NOA.

## Methods

### H&E, immunostaining, and imaging.

For H&E staining, testicular biopsy tissues were fixed in 4% paraformaldehyde, embedded in paraffin, and sectioned. Sections were deparaffinized, rehydrated, and stained with H&E. For immunochemistry analysis, sections of testicular tissue were stained with primary antibodies overnight at 4°C. The slides were then washed and incubated with a secondary antibody. Staining was visualized using 3,3′-diaminobenzidine, with hematoxylin used as the counterstain. Images were obtained on an Eclipse 80i microscope (Nikon). Immunofluorescence staining analysis on sections of testicular tissue was performed according to the protocol previously described by Nasr et al. (32).

For immunofluorescence staining of *Drosophila* testis, 3- to 5-day-old adult males were dissected in PBS and then fixed in 4% formaldehyde. They were then blocked for 1 hour in 5% BSA. The samples were incubated with primary antibodies overnight at 4°C, then incubated with secondary antibodies. Before finalizing, testis cells were stained with DAPI 1 g/mL for 15 minutes. The antibodies used are listed in Supplemental Table 1.

For the white light images of testes, 5-day-old adult males were dissected in PBS and placed on a glass slide containing a drop of PBS at 25°C, with images then obtained using a Nikon SMZ1500 microscope.

### WES and Sanger sequencing.

Genomic DNA was extracted using a HiPure Blood & Tissue DNA Kit (Magen, D3018) according to the manufacturer’s instructions. For whole-exome library construction, 500 ng to 1 μg DNA was ligated to a pair of adaptors after being randomly split into fragments of 200 bp or 300 bp on both sides. Targeted enrichment capture was achieved using Agilent Sure Select Human All Exon V6 + COSMIC (66M). The genomic region was captured using a HiSeq platform (Illumina) with paired-end sequencing. The trimmomatic tool (v0.36) was used to trim raw FASTQ data.

Sequencing read quality was inspected using FastQC software. Sequence reads were aligned to the human genome (hg19) using Burrows-Wheeler Aligner with default parameters. A Genome Analysis Toolkit (GATK) was used to realign reads around indels to reduce alignment artifacts and to recalibrate base quality scores. Duplicated reads were marked for removal, and the resulting BAM files were used for variant discovery. The single nucleotide variants and indel identifications were performed using GATK following its recommended best practices (33). Candidate gene variants for NOA meeting the following conditions were given preference: a) minor allele frequency < 0.01 in the 1000 Genomes, ESP6500, ExAC, and Genome Aggregation Databases; b) loss-of-function variants or potentially deleterious missense variants predicted by at least 2 of 4 software packages (SIFT, https://sift.bii.a-star.edu.sg/), PolyPhen-2 (http://genetics.bwh.harvard.edu/pph2/), MutationTaster (https://www.mutationtaster.org/), DDIG (https://sparks-lab.org/server/ddig/), or CRAVAT (http://hg19.cravat.us/CRAVAT/); c) gene variants that were identical in both brothers but differing from their father; and d) candidate genes highly expressed in the testis.

Specific PCR primers flanking the mutation for the candidate gene *RBBP7* were designed and used to amplify the regions *RBBP7* forward: 5′ AAACAGTGGACGAAGCACCA 3′ and *RBBP7* reverse: 5′ CACCCACCCCCAGTTGAAAT 3′. The amplified PCR products were analyzed using agarose gel electrophoresis to determine band size. Sanger sequencing was then carried out.

### PCR-RFLP.

To determine the *RBBP7* (chrX:16863960-/T) SNP in this family and in the 103 unrelated patients with NOA, we carried out the PCR-RFLP method. Primers were used to amplify *RBBP7* (forward primer: AAATAGCAAACAGTGGACGAA; reverse primer: TGAGGATAACATCATGCCGAT). The PCR conditions were as follows: denaturation at 94°C for 3 minutes followed by 94°C for 30 seconds, 58°C for 30 seconds, 72°C for 30 seconds for 35 cycles, with a final extension at 72°C for 5 minutes. The PCR products were treated with *Bgl* I (Takara, code 1020A) restriction enzyme. The PCR product of the wild-type *RBBP7* was cut into a 19 bp and a 207 bp fragment at 37°C, whereas the PCR product of the *RBBP7* mutation could not be cut. The resulting fragments were separated by electrophoresis in a 3% agarose gel containing 10 μg/mL ethidium bromide for 40 minutes at 100 V. The sizes of the resulting DNA fragments were estimated by comparison to that of a commercial DL1000 DNA marker (Takara, code 3591A).

### In silico analyses.

Human RBBP7 amino acid sequences were downloaded from the National Center for Biotechnology Information (https://www.ncbi.nlm.nih.gov/). The *Drosophila* Caf1-55 amino acid sequence was downloaded from Flybase (http://flybase.org/), and for the multiple amino acid sequence alignments, Clustal X 2.0 software was used. Results were highlighted using the DNAman software (version 9.0).

Structural analysis of the RBBP7 (NP_008999.2) variant was performed using the PDB database (https://www.rcsb.org/) based on the template of the structure (PDB code 7M3X and 3CFV), PyMol (https://pymol.org/2/), and UCSF ChimeraX software (https://www.cgl.ucsf.edu/chimerax). For analysis of the hydrophobic or hydrophilic scales of RBBP7 protein, we used the online tool Expasy server ProtScale module (https://web.expasy.org/protscale/). An improved deep learning–based modeling method, AlphaFold 2 (34), was used to accurately predict the *RBBP7* mutant protein structure, and ZDOCK server (http://zdock.umassmed.edu/) was used to analyze RBBP7 and BRCA1 docking.

### Western blot analysis.

Total protein extracted from GC-1/GC-2 cells or testis was prepared in lysis buffer (1× RIPA buffer: 50 mM Tris-HCl at pH 8.0, 150 mM NaCl, 1% IGEPAL CA-630, 0.5% sodium deoxycholate, 0.1% SDS) containing protease inhibitor cocktail (Roche) and quantified using a BCA assay (Beyotime). Equivalent amounts of total protein per sample were diluted with loading buffer, boiled for 5 minutes, and stored at –20°C until use. Samples were subjected to SDS-PAGE, transferred to a PVDF membrane, and immunoblotted at 4°C overnight with antibodies. The primary antibodies used were rabbit anti-RBBP7 (Abbexa, 141930, 1:200) and mouse anti-GAPDH (Good Here, 1:2,000). The secondary antibodies were HRP conjugated (1:5,000) (Supplemental Table 2). Blots were treated with ChemiLucent ECL detection reagents (MilliporeSigma), and protein bands were visualized using a Chemiluminescence Imaging System (Clinx Science Instruments).

### qRT-PCR.

Total RNA from cultured cells was extracted using TRIzol reagent (Thermo Fisher Scientific). cDNA was synthesized from the RNA samples using a First-Strand cDNA Synthesis Kit (Thermo Fisher Scientific). We used the Power SYBR Green PCR Master Mix (Thermo Fisher Scientific) to conduct RT-PCRs with GAPDH as a control. Real-time PCR was performed on an ABI 7900HT Fast Real-Time PCR machine (Thermo Fisher Scientific). The PCR primers used are as follows: *GAPDH* (forward primer: AGGTCGGTGTGAACGGATTTG, reverse primer: TGTAGACCATGTAGTTGAGGTCA) and *RBBP7* (forward primer: GAGCGTGTCATCAACGAAGAG, reverse primer: CCACTGAACGGTAAGACTGGG).

### Cell culture and cell proliferation assay.

GC-1 cells (of mouse spermatogonia cell line) and GC-2 cells (of mouse spermatocyte cell line) were purchased from Kunming Cell Bank of the Chinese Academy of Sciences (GC-1spg, KCB2016078YJ; GC-2spd, KCB 2016075YJ). The GC-1 and GC-2 cell lines were grown in incomplete medium consisting of DMEM containing 10% FBS (Gibco), 100 U/mL penicillin, and 100 μg/mL streptomycin (MilliporeSigma) at 37°C with 5% CO_2_. Specific siRNAs were designed using siDirect Ver2.0 (http://sidirect2.rnai.jp/) and transfected in GC-1 cells and GC-2 using riboFECT CP Reagent (Guangzhou RIBOBIO) following the manufacturer’s recommendations. The *RBBP7* siRNA sequences used were as follows: sense 5′ CCAUGAAGGAGAAGUGAAUTT 3′, antisense 5′ AUUCACUUCUCCUUCAUGGTT 3′. Negative control and *RBBP7* siRNA were all synthesized by GenePharma Inc.

GC-1 and GC-2 cells were transfected with siRNA for 24 hours. For cell growth curve analysis, we used a Cell Counting Kit-8 (Biosharp, BS350A) following the manufacturer’s instructions. The assay was performed in triplicate and the average was calculated.

### Flow cytometry.

A cell cycle staining kit (CCS012) and Annexin V-FITC/PI apoptosis kit (AP101) were purchased from MultiSciences Biotech Co., Ltd. and used for flow cytometry. GC-1 and GC-2 cells transfected with siRNA were seeded into 24-well plates at a cell density of 1 × 10^5^ per milliliter cell suspension. After incubation for 48 hours at 37°C and 5% CO_2_ saturated humidity, the cells were rinsed 3 times with PBS, then fixed. Centrifugations (5 minutes, 300*g*, room temperature, 3 times) were performed to remove the fixative and to wash the cells with PBS. Then 1 mL DNA Staining solution and 10 μL Permeabilization solution were added at 37°C, followed by placement in a water bath for 30 minutes. Cell cycle analysis was then conducted using flow cytometry (ACEA NovoCyte flow cytometer, ACEA Biosciences).

For the apoptotic assay, GC-1 and GC-2 cells transfected with siRNA were seeded into a 6-well plate at a cell density of 1 × 10^6^/mL. Cells were cultured for 48 hours in the incubator and collected, washed, and fixed in 5 μL Annexin V-FITC plus 10 μL PI Staining Solution. Flow cytometry was then used to detect cell apoptosis.

### Drosophila stocks and genetics.

Flies were reared on standard cornmeal medium at 25°C. The following strains were used: w^1118^, daughterless-Gal4, nos-Gal4, tj-Gal4; Bloomington *Drosophila* Stock Center: *UAS-Caf1-55-IR2* (catalog 34069), w[*];Caf1-55[p55-1]/TM3, Sb[1] (catalog 68168); *UAS-Caf1-55* (provided by Rongwen Xi, National Institute of Biological Sciences, Beijing, China); and Tsinghua Fly Center: *UAS-Caf1-55-IR1* (THU0589).

To generate *UAS-RBBP7*, *UAS-RBBP7*^Δ^, and *UAS-Caf1-55*^Δ^ transgenic flies, we amplified the full-length *RBBP7* cDNA, mutant *RBBP7* cDNA, and mutant *Caf1-55* cDNA and cloned each of them into separate pUAST-attb vectors. These constructs were then transformed into 25C6 line embryos using the standard P-element–mediated transgenesis protocol.

### Fertility test.

Five-day-old males were selected from different genotypes and arranged to mate with wild-type virgin females in small cages for 1 day before calculating the embryo hatch rate. Embryos were then collected on new apple juice agar plates for 4 hours and incubated at 25°C and 45%–70% humidity for 24 hours. Hatch rates were determined from the proportion of hatched eggs to total eggs. Under microscopy the hatched embryos could easily be distinguished from the unhatched eggs, showing shriveled and discarded egg shells in comparison with those with the appearance of plump grains of white rice. This experiment was repeated 3 times.

### Statistics.

Statistical data are presented as the mean ± SEM. Most experiments included at least 3 independent samples and were repeated at least 3 times. Two-tailed unpaired Student’s *t* tests were used to compare the results of 2 experimental groups, and for multiple comparisons, 1-way ANOVA was performed with GraphPad Prism (version 9.3.1). *P* values of less than 0.05 were considered statistically significant.

### Study approval.

All human studies were approved by the Ethical Committee of the Women’s Hospital, Zhejiang University School of Medicine (IRB-20220163-R). All participants signed a document of informed consent before participation in the study.

### Data availability.

All data contained in this study are available from the corresponding author upon reasonable request. Values for all data points found in graphs are in the Supporting Data Values file. The variation data reported in this paper have been deposited in the Genome Variation Map (35) in National Genomics Data Center, China National Center for Bioinformation/Beijing Institute of Genomics, Chinese Academy of Sciences, under accession number GVM000548 that is publicly accessible (http://bigd.big.ac.cn/gvm/getProjectDetail?project=GVM000548).

## Author contributions

JL and HZ planned and conducted experiments, drafted the initial manuscript, and revised the manuscript. The order of the co–first authors’ names was determined alphabetically. JH performed experiments and acquired and analyzed data. JC conducted experiments. FZ provided samples and reagents. XY provided reagents and reviewed the manuscript. FJ conceptualized the research studies and reviewed the manuscript. YX conceptualized and designed the research studies and reviewed and revised the manuscript.

## Supplementary Material

Supplemental data

Supporting data values

## Version 1 10/16/2023

Electronic publication

## References

[1] Pan MM (2018). Male infertility diagnosis and treatment in the era of in vitro fertilization and intracytoplasmic sperm injection. Med Clin North Am.

[2] Weedin JW (2011). Early versus late maturation arrest: reproductive outcomes of testicular failure. J Urol.

[3] Ishikawa T (2004). Clinical and hormonal findings in testicular maturation arrest. BJU Int.

[4] Xu X (2003). Impaired meiotic DNA-damage repair and lack of crossing-over during spermatogenesis in BRCA1 full-length isoform deficient mice. Development.

[5] Li S (2022). Cancer risks associated with *BRCA1* and *BRCA2* pathogenic variants. J Clin Oncol.

[6] Ji ZY (2021). Novel hemizygous mutations of *TEX11* cause meiotic arrest and non-obstructive azoospermia in Chinese Han population. Front Genet.

[7] Yatsenko AN (2015). X-linked TEX11 mutations, meiotic arrest, and azoospermia in infertile men. N Engl J Med.

[8] Vockel M (2021). The X chromosome and male infertility. Hum Genet.

[9] Riera-Escamilla A (2022). Large-scale analyses of the X chromosome in 2,354 infertile men discover recurrently affected genes associated with spermatogenic failure. Am J Hum Genet.

[10] Murzina NV (2008). Structural basis for the recognition of histone H4 by the histone-chaperone RbAp46. Structure.

[11] Loyola A, Almouzni G (2004). Histone chaperones, a supporting role in the limelight. Biochim Biophys Acta.

[12] Chen GC (2001). Rb-associated protein 46 (RbAp46) inhibits transcriptional transactivation mediated by BRCA1. Biochem Biophys Res Commun.

[13] Balboula AZ (2014). Knockdown of RBBP7 unveils a requirement of histone deacetylation for CPC function in mouse oocytes. Cell Cycle.

[14] Furuyama T (2006). Chaperone-mediated assembly of centromeric chromatin in vitro. Proc Natl Acad Sci U S A.

[15] Wen P (2012). The biological function of the WD40 repeat-containing protein p55/Caf1 in Drosophila. Dev Dyn.

[16] Colecchia M (2007). Leydig cell tumor and hyperplasia - A review. Anal Quant Cytol Histol.

[17] Zhang BB (2021). Novel loss-of-function variants in DNAH17 cause multiple morphological abnormalities of the sperm flagella in humans and mice. Clin Genet.

[19] Zhao W (2019). The BRCA tumor suppressor network in chromosome damage repair by homologous recombination. Annu Rev Biochem.

[20] Coutelis JB (2008). The myosin ID pathway and left-right asymmetry in Drosophila. Genesis.

[21] Shivdasani AA, Ingham PW (2003). Regulation of stem cell maintenance and transit amplifying cell proliferation by tgf-beta signaling in Drosophila spermatogenesis. Curr Biol.

[22] Jan SZ (2018). Distinct prophase arrest mechanisms in human male meiosis. Development.

[23] Schmidberger JW (2016). The MTA1 subunit of the nucleosome remodeling and deacetylase complex can recruit two copies of RBBP4/7. Protein Sci.

[24] Yeh H-H (2015). Ras induces experimental lung metastasis through up-regulation of RbAp46 to suppress RECK promoter activity. BMC Cancer.

[25] Schlecht U (2004). Expression profiling of mammalian male meiosis and gametogenesis identifies novel candidate genes for roles in the regulation of fertility. Mol Biol Cell.

[26] Adamczewska D (2022). The fate of leydig cells in men with spermatogenic failure. Life (Basel).

[27] Naughton CK (1998). Leydig cell hyperplasia. Br J Urol.

[28] Pitteloud N (2008). , et al. Inhibition of luteinizing hormone secretion by testosterone in men requires aromatization for its pituitary but not its hypothalamic effects: evidence from the tandem study of normal and gonadotropin-releasing hormone-deficient men. J Clin Endocrinol Metab.

[29] Dabbous Z, Atkin SL (2018). Hyperprolactinaemia in male infertility: Clinical case scenarios. Arab J Urol.

[30] Carter JN (1978). Prolactin-screening tumors and hypogonadism in 22 men. N Engl J Med.

[32] Nasr SH (2006). Immunofluorescence on pronase-digested paraffin sections: a valuable salvage technique for renal biopsies. Kidney Int.

[33] McKenna A (2010). The Genome Analysis Toolkit: a MapReduce framework for analyzing next-generation DNA sequencing data. Genome Res.

[34] Jumper J (2021). Highly accurate protein structure prediction with AlphaFold. Nature.

[35] Li CP (2021). Genome Variation Map: a worldwide collection of genome variations across multiple species. Nucleic Acids Res.

